# Exosomal miR-4488 and miR-1273g-5p inhibit the epithelial-mesenchymal transition of transforming growth factor β2-mediated retinal pigment epithelial cells by targeting ATP-binding cassette A4

**DOI:** 10.1080/21655979.2021.1987068

**Published:** 2021-12-25

**Authors:** Hongtao Dong, Menghua Wang, Qiuming Li

**Affiliations:** Department of Ophthalmology, The First Affiliated Hospital of Zhengzhou University, Zhengzhou City, Henan, China

**Keywords:** Proliferative vitreoretinopathy, exosomal, miR-4488, miR-1273g-5p, ABCA4

## Abstract

Exosomal microRNAs (miRNAs) have been shown to be involved in the regulation of many disease progression, including proliferative vitreoretinopathy (PVR). However, the roles of exosomal miR-4488 and miR-1273 g-5p in PVR progression have not been demonstrated. Transforming growth factor β2 (TGF-β2)-induced ARPE-19 cells were used to stimulate the epithelial-mesenchymal transition (EMT) of cells. Exosomes derived from TGF-β2-induced ARPE-19 cells were identified by transmission electron microscopy and nanoparticle tracking analysis. The expression levels of miR-4488, miR-1273 g-5p and ATP-binding cassette A4 (ABCA4) were measured by quantitative real-time PCR. The promotion levels of exosomes markers, EMT markers, apoptosis markers and ABCA4 were determined by western blot analysis. The migration, invasion and apoptosis of cells were determined by transwell assay, wound healing assay and flow cytometry. Our data showed that miR-4488 and miR-1273 g-5p were lowly expressed in TGF-β2-induced ARPE-19 cells. Overexpressed exosomal miR-4488 and miR-1273 g-5p could inhibit the EMT, migration, invasion, and promote apoptosis in TGF-β2-induced ARPE-19 cells. In addition, ABCA4 was a target of miR-4488 and miR-1273 g-5p. Overexpressed ABCA4 also could reverse the negatively regulation of exosomal miR-4488 and miR-1273 g-5p on the EMT, migration, and invasion of TGF-β2-induced ARPE-19 cells. In conclusion, our data showed that exosomal miR-4488 and miR-1273 g-5p could inhibit TGF-β2-stimulated EMT in ARPE-19 cells through targeting ABCA4.

## Introduction

Proliferative vitreoretinopathy (PVR) is a related disease caused by the proliferation of vitreous or retinal surface cells to form fibrous membrane, which then contracts and stretches [1,2]. The pathogenesis of PVR is very complicated, and retinal pigment epithelium (RPE) cells are currently recognized as the cell component that plays an important role in PVR development [3,4]. RPE cells detached from the basement membrane can activate fibroblast-like active cells for migration and epithelial-mesenchymal transition (EMT), and ultimately cause PVR [5,6]. In addition to surgery and drug therapy, molecular targeted therapy has also achieved good results in PVR [7,8]. Therefore, elucidating the molecular mechanisms that affect the biological functions of RPE cells may provide a theoretical basis for mitigating PVR progression. Transforming growth factor β2 (TGF-β2), a multifunctional cytokine, can induce the EMT process of RPE cells, which is widely used to construct PVR cell models *in vitro* [9,10].

Exosomes are tiny membrane vesicles (30–150 nm) that can be secreted by most cells, and contain a variety of non-coding RNAs including microRNAs (miRNAs) [11,12]. Exosomes are an important medium that mediates communication between cells, which can transmit specific molecular information and then change the biological functions of recipient cells [13,14]. For example, exosome miR-146a could enhance the chemosensitivity of ovarian cancer cells [15], and exosome miR-22-3p could promote endometriosis progression through increasing cell proliferation and migration [16]. Recently, Zhang *et al*. found that there were a total of 34 differentially expressed miRNAs in ARPE-19 cells treated with or without TGF-β2 used high-throughput sequencing [17]. Among them, we noted that miR-4488 and miR-1273 g-5p were significantly lower expressed in TGF-β2-induced ARPE-19 cells, but their roles in the EMT process of REP cells had not been investigated.

ATP-binding cassette A4 (ABCA4), a member of the ATP-binding cassette transporter subfamily ABCA, is expressed in cones and rods of vertebrates [18]. The abnormally expressed ABCA4 can lead to the accumulation of retinal toxic products, and then lead to the occurrence of retinal degeneration diseases [19,20]. Studies had confirmed that ABCA4 was remarkably upregulated in PVR tissues, and its knockdown had an inhibition effect on RPE cells proliferation and migration [21]. The above evidence confirmed that ABCA4 might be a key regulator for PVR progression.

Our study aimed to reveal the role and mechanism of exosomal miR-4488 and miR-1273 g-5p in the progression of PVR. Through online software, we found that miR-4488 and miR-1273 g-5p could interact with ABCA4. Therefore, we proposed the hypothesis that exosomal miR-4488 and miR-1273 g-5p might mediate PVR progression by targeting ABCA4. Our research goal is to provide potential molecular targets for the treatment of PVR.

## Materials and methods

### Cell culture and TGF-β2 treatment

Human RPE cells (ARPE-19) were obtained from ATCC (Manassas, VA, USA) and were cultured in DMEM/F12 medium (Gibco, Grand Island, NY, USA) supplemented with 10% FBS (Gibco) and 1% Penicillin/Streptomycin Solution (Invitrogen, Carlsbad, CA, USA). TGF-β2 treatment could be performed when the cell confluences reached 80%. Celle were treated with human recombinant TGF-β2 (10 ng/mL; R&D, Minneapolis, MN, USA) for 24 h as the previously described [22].

### Cell transfection

MiR-4488 mimic, miR-1273 g-5p mimic, pcDNA ABCA4 overexpression vector and their mitched negative controls were constructed by Ribobio (Guangzhou, China). Cells with 60% confluences could be transfected using Lipofectamine 3000 Reagent (Invitrogen). After transfection for 24 h, cells were treated with TGF-β2 for 24 h and then collected for function experiments.

### Quantitative real-time PCR (qRT-PCR)

Total RNA was extracted from ARPE-19 cells using TRIzol reagent (Invitrogen). Then, cDNA was synthesized using PrimeScript RT reagent Kit (TaKaRa, Dalian China). PCR was performed with SYBR Green (Solarbio, Beijing, China). 2^−ΔΔCT^ method was used to calculate relative expression with U6 or β-actin as internal control as the previously described [17]. The primer sequences were shown as Table 1.

**Table 1.** The primer sequences used for qRT-PCR

Name		Primers for PCR (5ʹ-3ʹ)
miR-4488	Forward	AGGGGGCGGGCUCCGGCG
	Reverse	GAACATGTCTGCGTATCTC
miR-1273 g-5p	Forward	GCCGAGGGTGGTTGAGGCTGCA
	Reverse	CAGTGCGTGTCGTGGAGT
ABCA4	Forward	CATCTTGGCAAGGGTATATCGAG
	Reverse	CTGCAATTCTCTCCGGGTGAG
U6	Forward	CTCGCTTCGGCAGCACA
	Reverse	AACGCTTCACGAATTTGCGT
β-actin	Forward	CTTCGCGGGCGACGAT
	Reverse	CCACATAGGAATCCTTCTGACC

### Exosome isolation

ExoQuick (System Biosciences, Palo Alto, CA, USA) was used to extract exosomes from TGF-β2-treated ARPE-19 cells as the previously described [17]. According to the kit instructions, the cell supernatant was centrifuged and incubated with ExoQuick solution, and then centrifuged again to obtain exosome pellets.

### Identification of exosomes

The morphology and size of exosomes were analyzed using transmission electron microscopy (TEM) and nanoparticle tracking analysis (NTA) as the previously described [17].

### Western blot (WB) analysis

As the previously described [23], RIPA Lysis buffer (Beyotime, Shanghai, China) was used to extract total protein. Equal amount of protein was separated using 10% SDS-PAGE gel and then electro-transferred onto PVDF membranes (Invitrogen). The membranes were blocked with 5% nonfat milk followed by incubated overnight with primary antibodies at 4°C, including CD9 (1:2,000, ab92726, Abcam, Cambridge, MA, USA), CD63 (1:1,000, ab134045, Abcam), CD81 (1:2,000, ab109201, Abcam), ZO-1 (1:1,000, ab216880, Abcam), occludin (1:250, ab31721, Abcam), E-cadherin (1:10,000, ab40772, Abcam), α-SMA (1:1,000, ab5694, Abcam), Bcl-2 (1:1,000, ab194583, Abcam), Bax (1:1,000, ab32503, Abcam), cleaved caspase 3 (1:1,000, ab2302, Abcam), and β-actin (1:1,000, ab5694, Abcam). After further incubated with secondary antibody (1:50,000, ab205718, Abcam), the protein signals were visualized using Enhanced Chemiluminescence Detection Kit (Vazyme, Nanjing, China).

### Transwell assay

As the previously described [24], 24-well transwell chambers (8 µm, BD Biosciences, Heidelberg, Germany) was used to detect cell migration, which was pre-coated with a Matrigel (BD Biosciences) for measuring cell invasion. ARPE-19 cells (1 × 10^5^ cells) were added into the upper of chambers with serum-free medium. The lower chambers were filled with 600 µL serum medium. After cultured for 24 h at 37°C, the cells were fixed with paraformaldehyde and stained with crystal violet. The number of cells was counted to calculate cell migration ability and invasion ability (%).

### Wound healing assay

As the previously described [24], ARPE-19 cells were seeded into 6-well plates. A 200 μL pipette tip was used to create a wound in the cell layer when the cells reached 90% confluence. After that, the cells were replaced with serum-free medium and incubated for 24 h at 37°C. The wound area was then photographed under a microscope (40 ×) at 0 h and 24 h, and the percentage wound closure was calculated.

### Flow cytometry

As the previously described [25], Annexin V-FITC/PI Apoptosis Detection Kit (Vazyme) was used to detect cell apoptosis. Briefly, ARPE-19 cells were re-suspended with 1 × Binding Buffer followed by treated with Annexin V-FITC solution and PI staining solution. Cell apoptosis rate was analyzed by CytoFLEX flow cytometer.

### Dual-luciferase reporter assay

According to the prediction results by DianaTools = microT_CDS software, the sequences of ABCA4 3ʹUTR containing the binding sites of miR-4488 or miR-1273 g-5p were inserted into pGL3 vectors, building the ABCA4-1 3ʹUTR WT or ABCA4-2 3ʹUTR WT vector. Besides, the fragments of ABCA4 3ʹUTR containing the mutant sites of miR-4488 or miR-1273 g-5p were generated in the same way, namely as the ABCA4-1 3ʹUTR MUT or ABCA4-2 3ʹUTR MUT vectors. As the previously described [25], 293 T cells were co-transfected with vectors and miR-4488 mimic or miR-1273 g-5p mimic for 48 h. The luciferase activities were examined by Dual-Luciferase Reporter Assay Kit (Promega, Madison, WI, USA).

### RIP assay

According to the instructions of Magna RIP Kit (Millipore, Billerica, MA, USA), ARPE-19 cells were treated with RIP buffer, and the cell lysates were incubated with magnetic beads pre-coated with Anti-AGO2 or Anti-IgG as the previously described [25]. The enrichment of miR-4488, miR-1273 g-5p and ABCA4 was detected by qRT-PCR.

## Statistical analysis

All statistical analysis was performed in GraphPad Prism 6.0 software and presented as mean ± SD from 3 independent experiments. Differences were analyzed using Student’s *t*-test or ANOVA followed by Tukey’s post-hoc test. Statistical significance was accepted at *P* < 0.05.

## Results

Our study aims to explore the role of miR-4488 and miR-1273 g-5p in PVR and reveal their underlying molecular mechanism. We conducted this study around the hypothesis that exosomal miR-4488 and miR-1273 g-5p mediated PVR progression by regulating ABCA4. First, we determined that exosomal miR-4488 and miR-1273 g-5p were involved in the regulation on TGF-β2-treated ARPE-19 cell progression. Then, the interaction between ABCA4 and miR-4488 or miR-1273 g-5p was determined by software prediction and experimental verification. Finally, rescue experiments were performed to confirm that exosomal miR-4488 and miR-1273 g-5p mediated TGF-β2-treated ARPE-19 cell progression by regulating ABCA4.

### Exosomes mediated the transport of miR-4488 and miR-1273 g-5p in TGF-β2-treated ARPE-19 cells

**Figure 1.**
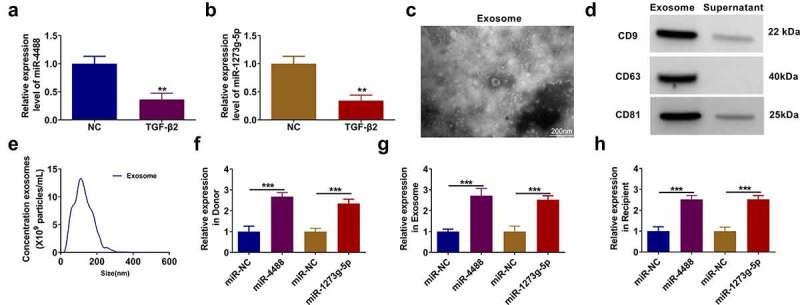
**Exosomes mediated the transport of miR-4488 and miR-1273 g-5p in TGF-β2-treated ARPE-19 cells**. (a-b) The expression of miR-4488 and miR-1273 g-5p in ARPE-19 cells treated with or without TGF-β2 was measured by qRT-PCR. (c) The morphology of exosomes from TGF-β2-treated ARPE-19 cells was analyzed using TEM. (d) The expression of exosome markers (CD9, CD63 and CD81) was detected by WB analysis. (e) The size of exosomes was analyzed by NTA. (f) Donor ARPE-19 cells were transfected with miR-4488 mimic or miR-1273 g-5p mimic followed by treated with TGF-β2. The expression of miR-4488 and miR-1273 g-5p was detected by qRT-PCR. (g) Exosomes were isolated from donor ARPE-19 cells transfected with miR-4488 mimic or miR-1273 g-5p mimic followed by treated with TGF-β2. The expression of miR-4488 and miR-1273 g-5p in exosomes was determined by qRT-PCR. (h) Recipient ARPE-19 cells were treated with exosomes followed by treated with TGF-β2. The expression of miR-4488 and miR-1273 g-5p were detected by qRT-PCR. **P < 0.01, ***P < 0.001

In TGF-β2-treated ARPE-19 cells, we discovered that miR-4488 and miR-1273 g-5p were lowly expressed compared to the non-treated cells (Figure 1a-b). After isolated the exosomes from TGF-β2-treated ARPE-19 cells, the morphology of exosomes was analyzed using TEM (Figure 1c). Besides, the expression of exosome markers (CD9, CD63 and CD81) was detected, and the results showed that they were overexpressed in the exosomes from TGF-β2-treated ARPE-19 cells (Figure 1d). Furthermore, the size of exosomes was analyzed by NTA (Figure 1e). To explore whether exosomes mediated the transport of miR-4488 and miR-1273 g-5p, donor ARPE-19 cells were transfected with the mimic of miR-4488 and miR-1273 g-5p followed by treated with TGF-β2. After confirmed that the mimics could markedly increase the expression of miR-4488 and miR-1273 g-5p (figure 1f), we isolated the exosomes from the donor cells. In the exosomes (named as Exo-miR-NC, Exo-miR-4488 and Exo-miR-1273 g-5p), we found that miR-4488 and miR-1273 g-5p expression was significantly enhanced (Figure 1g). Then, the exosomes were incubated with the recipient TGF-β2-treated ARPE-19 cells for 24 h. The detection results of miR-4488 and miR-1273 g-5p showed that the expression levels of miR-4488 and miR-1273 g-5p also were remarkably increased in the recipient TGF-β2-treated ARPE-19 cells co-cultured with Exo-miR-4488 and Exo-miR-1273 g-5p, respectively (Figure 1h). Above all, we confirmed that exosomes mediated the transport of miR-4488 and miR-1273 g-5p in TGF-β2-treated ARPE-19 cells.

### Exosomal miR-4488 inhibited the EMT, migration and invasion of TGF-β2-induced ARPE-19 cells

**Figure 2.**
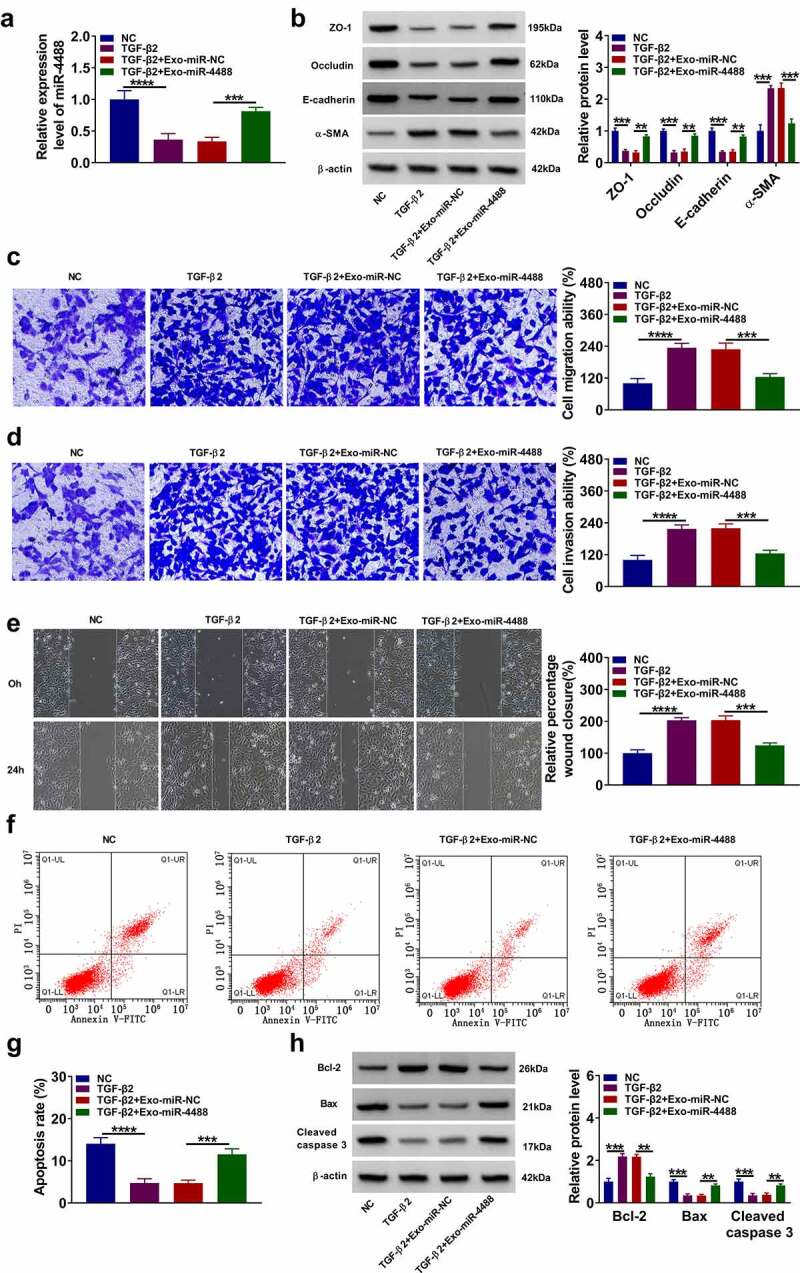
**Exosome miR-4488 inhibited the EMT, migration and invasion of TGF-β2-induced ARPE-19 cells**. ARPE-19 cells were treated with exosomes (Exo-miR-NC or Exo-miR-4488) followed by treated with or without TGF-β2. Non treated cells were used as normal control (NC). (a) The expression of miR-4488 was measured by qRT-PCR. (b) The protein levels of ZO-1, occludin, E-cadherin and α-SMA were detected by WB analysis. (c-d) The migration and invasion abilities were measured by transwell assay. (e) Wound healing assay was used to assess the migration of cells. (f-g) The apoptosis rate of cells was determined by flow cytometry. (h) The protein levels of Bcl-2, Bax and cleaved caspase 3 were assessed by WB analysis. **P < 0.01, ***P < 0.001, ****P < 0.0001

To explore the role of exosomal miR-4488 in PVR progression, ARPE-19 cells were treated with Exo-miR-4488 for 24 h followed by treated with TGF-β2 for 24 h. The inhibition effect of TGF-β2 on miR-4488 expression could be promoted by the addition of Exo-miR-4488 (Figure 2a). TGF-β2 treatment reduced the protein levels of ZO-1, occludin and E-cadherin, while increased the protein level of α-SMA in ARPE-19 cells. However, this effect could be reversed by overexpressing exosomal miR-4488 (Figure 2b). Moreover, TGF-β2 could promote the migration and invasion abilities, as well as enhance the wound closure of ARPE-19 cells, while overexpression of exosomal miR-4488 also could inhibit cell migration and invasion (Figure 2c-e). Flow cytometry results indicated that Exo-miR-4488 promoted the apoptosis rate of TGF-β2-treated ARPE-19 cells (figure 2f-g). Also, TGF-β2 treatment increased the Bcl-2 protein level, decreased the Bax protein level, and inhibited the cleaved caspase 3 protein level, while these effects could be abolished by the addition of Exo-miR-4488 (Figure 2h). Therefore, we confirmed that exosomal miR-4488 might inhibit PVR progression.

### MiR-4488 directly targeted ABCA4

**Figure 3.**
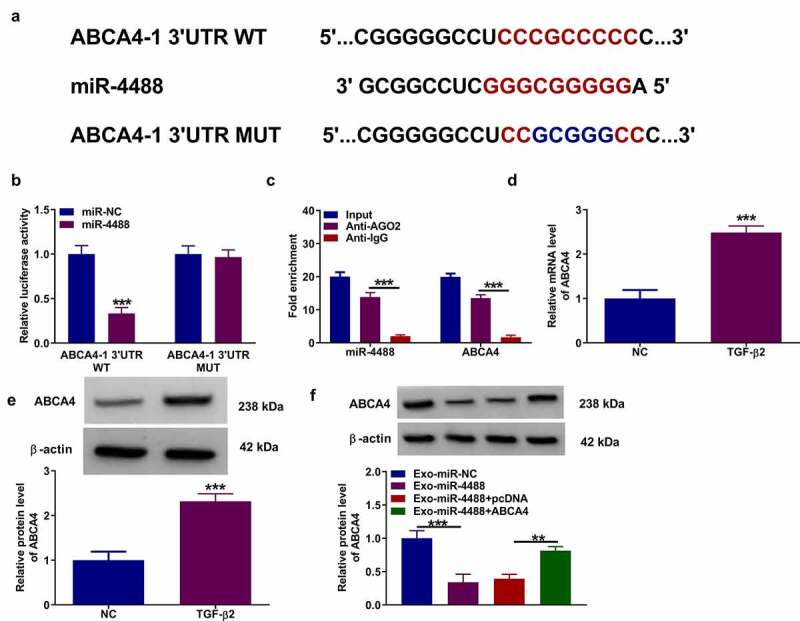
**MiR-4488 directly targeted ABCA4**. (a) The binding sites and mutant sites between ABCA4 3ʹUTR and miR-4488 were shown. Dual-luciferase reporter assay (b) and RIP assay (c) were used to confirm the interaction between ABCA4 and miR-4488. (d-e) The mRNA and protein levels of ABCA4 in ARPE-19 cells treated with or without TGF-β2 was determined by qRT-PCR and WB analysis. (f) ARPE-19 cells were transfected with or without pcDNA or pcDNA ABCA4 overexpression vector, and then treated with or without Exo-miR-4488 or Exo-miR-NC followed by treated with TGF-β2. The protein level of ABCA4 was detected by WB analysis. **P < 0.01, ***P < 0.001

Using the DianaTools = microT_CDS software, we found that there had binding sites between miR-4488 and ABCA4 3ʹUTR. According to the binding sites, we constructed the ABCA4-1 3ʹUTR WT/MUT vectors (Figure 3a). Dual-luciferase reporter assay results suggested that miR-4488 mimic could reduce the luciferase activity of ABCA4-1 3ʹUTR WT vector without affecting that of the ABCA4-1 3ʹUTR MUT vector (Figure 3b). Also, RIP assay results showed that miR-4488 and ABCA4 could be enriched in Anti-AGO2 (Figure 3c). In TGF-β2-treated ARPE-19 cells, we confirmed that ABCA4 expression was upregulated at the mRNA level and protein level (Figure 3d-e). Besides, we found that Exo-miR-4488 could reduce ABCA4 protein level in TGF-β2-induced ARPE-19 cells, while this effect could be reversed by the transfection of pcDNA ABCA4 overexpression vector (figure 3f). These data showed that miR-4488 negatively regulated ABCA4 expression.

### ABCA4 reversed the effect of exosomal miR-4488 on the EMT, migration and invasion of TGF-β2-induced ARPE-19 cells

**Figure 4.**
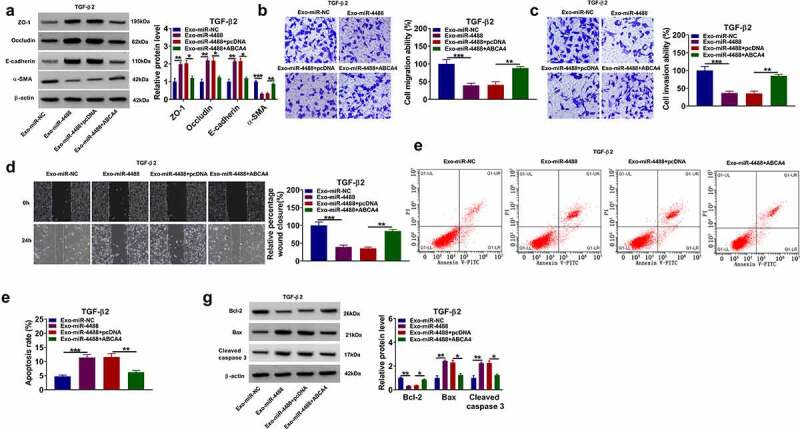
**ABCA4 reversed the effect of exosomal miR-4488 on the EMT, migration and invasion of TGF-β2-induced ARPE-19 cells**. ARPE-19 cells were transfected with or without pcDNA or pcDNA ABCA4 overexpression vector and then treated with or without Exo-miR-4488 or Exo-miR-NC followed by treated with TGF-β2. (a) WB analysis was used to detect the protein levels of ZO-1, occludin, E-cadherin and α-SMA. (b-c) Transwell assay was performed to measure cell migration and invasion abilities. (d) The migration of cells was evaluated using wound healing assay. (e-f) The apoptosis rate of cells was assessed using flow cytometry. (g) WB analysis was utilized to determine the protein levels of Bcl-2, Bax and cleaved caspase 3. *P < 0.05, **P < 0.01, ***P < 0.001

Then, we assessed the EMT, migration and invasion of TGF-β2-induced ARPE-19 cells treated with Exo-miR-4488 and transfected with pcDNA ABCA4 overexpression vector. Our data showed that the enhancing effect of exosomal miR-4488 on the protein levels of ZO-1, occludin and E-cadherin, as well as the suppressive effect on the protein level of α-SMA could be reversed by overexpressing ABCA4 in TGF-β2-induced ARPE-19 cells (Figure 4a). Besides, the inhibition effect of exosomal miR-4488 on the migration ability, invasion ability, and the wound closure rate in TGF-β2-induced ARPE-19 cells also could be abolished by ABCA4 overexpression (Figure 4b-d). Overexpression of ABCA4 reduced the apoptosis increased by exosomal miR-4488 in TGF-β2-induced ARPE-19 cells (Figure 4e). The negatively regulation of exosomal miR-4488 on Bcl-2 protein expression and the positively regulation on Bax and cleaved caspase 3 protein expression also could be reversed by overexpressing ABCA4 (figure 4f). These results revealed that exosomal miR-4488 targeted ABCA4 to inhibit PVR progression.

### Exosomal miR-1273 g-5p had an inhibition on the EMT, migration and invasion of TGF-β2-induced ARPE-19 cells

**Figure 5.**
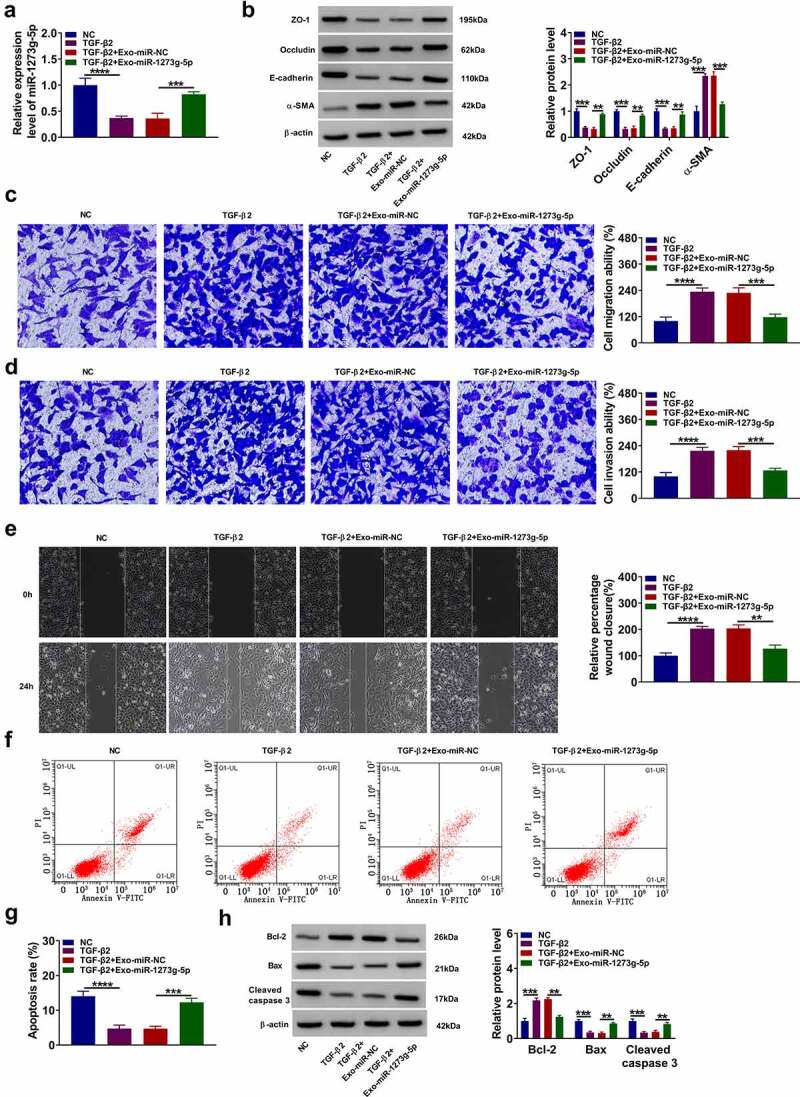
**Exosomal miR-1273 g-5p had an inhibition on the EMT, migration and invasion of TGF-β2-induced ARPE-19 cells**. ARPE-19 cells were treated with exosomes (Exo-miR-NC or Exo-miR-1273 g-5p) followed by treated with or without TGF-β2. Non treated cells were used as normal control (NC). (a) The miR-1273 g-5p expression was detected by qRT-PCR. (b) The protein levels of ZO-1, occludin, E-cadherin and α-SMA were determined using WB analysis. (c-d) Transwell assay was utilized to test cell migration and invasion abilities. (e) Wound healing assay was performed to measure cell migration. (f-g) Cell apoptosis rate was evaluated by flow cytometry. (h) The protein levels of Bcl-2, Bax and cleaved caspase 3 were analyzed by WB analysis. *P < 0.05, **P < 0.01, ***P < 0.001, ****P < 0.0001

In TGF-β2-induced ARPE-19 cells treated with Exo-miR-1273 g-5p, we evaluated the biological functions of cells to assess the role of miR-1273 g-5p in PVR progression. The treatment of Exo-miR-1273 g-5p could promote miR-1273 g-5p expression in TGF-β2-induced ARPE-19 cells (Figure 5a). Also, Exo-miR-1273 g-5p enhanced the protein levels of ZO-1, occludin and E-cadherin, while decreased the protein level of α-SMA in TGF-β2-induced ARPE-19 cells (Figure 5b). In addition, Exo-miR-1273 g-5p reduced the migration ability, invasion ability, and the wound closure of TGF-β2-induced ARPE-19 cells (Figure 5c-e). The apoptosis rate of TGF-β2-treated ARPE-19 cells also could be promoted by the addition of Exo-miR-1273 g-5p (figure 5f). Furthermore, Exo-miR-1273 g-5p decreased the Bcl-2 protein level, while promoted the Bax and cleaved caspase 3 protein levels in TGF-β2-treated ARPE-19 cells (Figure 5g). All data showed that exosomal miR-1273 g-5p could suppress PVR progression.

### ABCA4 also could be targeted by miR-1273 g-5p

**Figure 6.**
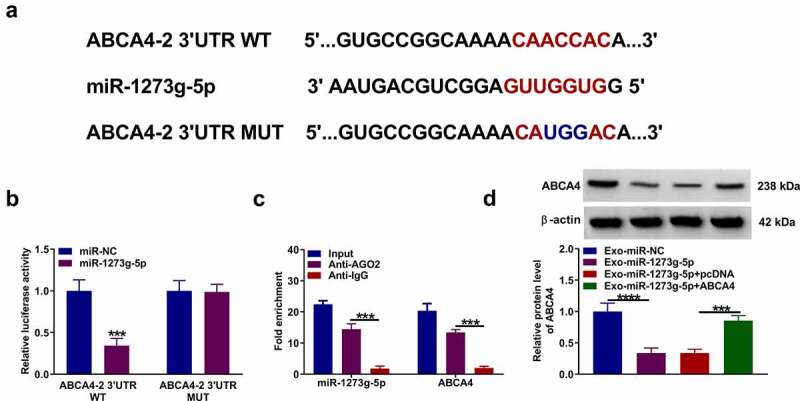
**ABCA4 also could be targeted by miR-1273 g-5p**. (a) The binding sites and mutant sites between ABCA4 3ʹUTR and miR-1273 g-5p were shown. Dual-luciferase reporter assay (b) and RIP assay (c) were used to confirm the interaction between ABCA4 and miR-1273 g-5p. (d) ARPE-19 cells were transfected with or without pcDNA or pcDNA ABCA4 overexpression vector and then treated with or without Exo-miR-1273 g-5p or Exo-miR-NC followed by treated with TGF-β2. WB analysis was used to measure the protein level of ABCA4. ***P < 0.001, ****P < 0.0001

According the prediction results of DianaTools = microT_CDS software, we confirmed that miR-1273 g-5p also could bind with the 3ʹUTR of ABCA4. The ABCA4-2 3ʹUTR WT/MUT vectors were constructed basing on the binding sites between ABCA4 3ʹUTR and miR-1273 g-5p (Figure 6a). Dual-luciferase reporter assay and RIP assay were used to confirm the interaction between ABCA4 and miR-1273 g-5p. The results suggested that miR-1273 g-5p mimic could inhibit the luciferase activity of ABCA4-2 3ʹUTR WT vector (Figure 6b), and both of them were enriched in Anti-AGO2 (Figure 6c). In addition, we found that Exo-miR-1273 g-5p could decrease ABCA4 protein expression in TGF-β2-treated ARPE-19 cells, while this effect could be reversed by pcDNA ABCA4 overexpression vector (Figure 6d). These data revealed that miR-1273 g-5p could target ABCA4.

### Exosomal miR-1273 g-5p regulated the EMT, migration and invasion of TGF-β2-induced ARPE-19 cells by targeting ABCA4

**Figure 7.**
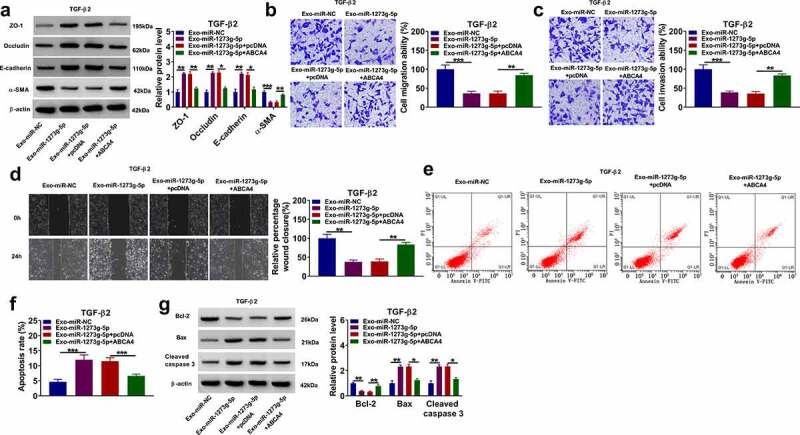
**Exosomal miR-1273 g-5p regulated the EMT, migration, invasion and apoptosis of TGF-β2-induced ARPE-19 cells by targeting ABCA4**. ARPE-19 cells were transfected with or without pcDNA or pcDNA ABCA4 overexpression vector and then treated with or without Exo-miR-1273 g-5p or Exo-miR-NC followed by treated with TGF-β2. (a) The protein levels of ZO-1, occludin, E-cadherin and α-SMA were measured by WB analysis. (b-c) Transwell assay was performed to detect cell migration and invasion abilities. (d) Wound healing assay was used to detect the migration of cells. (e-f) Cell apoptosis rate was analyzed using flow cytometry. (g) WB analysis was used to measure the protein levels of Bcl-2, Bax and cleaved caspase 3. *P < 0.05, **P < 0.01, ***P < 0.001

In TGF-β2-induced ARPE-19 cells treated with Exo-miR-1273 g-5p and transfected with pcDNA ABCA4 overexpression vector, we found that ABCA4 overexpression reversed the promotion effect of Exo-miR-1273 g-5p on the protein levels of ZO-1, occludin and E-cadherin, as well as the inhibition effect on the protein level of α-SMA (Figure 7a). Besides, the enhancing effect of Exo-miR-1273 g-5p on the migration ability, invasion ability, and the wound closure rate in TGF-β2-induced ARPE-19 cells also could be abolished by overexpressing ABCA4 (Figure 7b-d). In addition, the regulation of Exo-miR-1273 g-5p on the apoptosis rate and the protein levels of Bcl-2, Bax and cleaved caspase 3 also could be reversed by overexpression of ABCA4 in TGF-β2-induced ARPE-19 cells (Figure 7e-f). Hence, our data confirmed that exosomal miR-1273 g-5p indeed targeted ABCA4 to regulate PVR progression.

### Overexpressed ABCA4 promoted EMT, migration and invasion in TGF-β2-induced ARPE-19 cells

**Figure 8.**
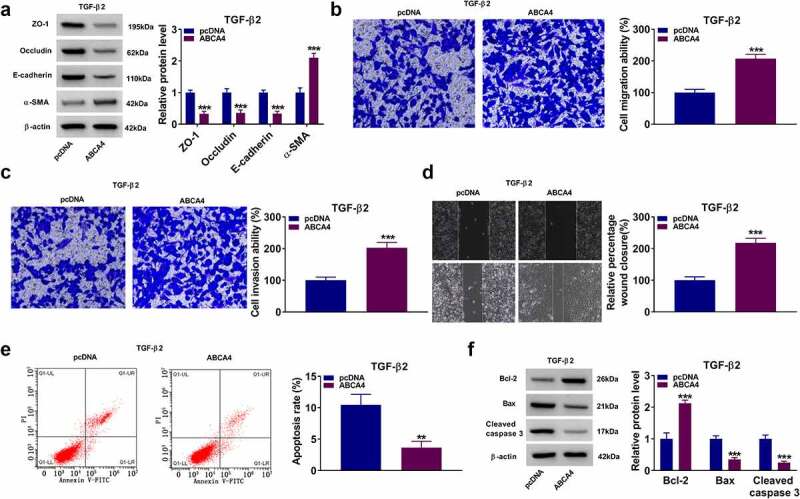
**ABCA4 overexpression promoted EMT, migration and invasion in TGF-β2-induced ARPE-19 cells**. ARPE-19 cells were transfected with pcDNA or pcDNA ABCA4 overexpression vector followed by treated with TGF-β2. (a) WB analysis was performed to detect the protein levels of ZO-1, occludin, E-cadherin and α-SMA. (b-c) Cell migration and invasion abilities were determined by transwell assay. (d) Wound healing assay was utilized for measuring the migration of cells. (e) Flow cytometry was used to examine cell apoptosis rate. (f) WB analysis was performed to analyze the protein levels of Bcl-2, Bax and cleaved caspase 3. **P < 0.01, ***P < 0.001

To further confirm the role of ABCA4 in PVR progression, ARPE-19 cells were transfected with pcDNA ABCA4 overexpression vector and then treated with TGF-β2. Our data showed that overexpressed ABCA4 could decrease the protein levels of ZO-1, occludin and E-cadherin, while increase the protein level of α-SMA in TGF-β2-induced ARPE-19 cells (Figure 8a). Also, ABCA4 overexpression promoted the migration ability, invasion ability, and the wound closure of TGF-β2-induced ARPE-19 cells (Figure 8b-d). Furthermore, ABCA4 overexpression also inhibited apoptosis rate, enhanced Bcl-2 protein level, suppressed Bax level, and reduced cleaved caspase-3 level in TGF-β2-induced ARPE-19 cells (Figure 8e-f). These data confirmed that ABCA4 overexpression could promote EMT, migration, invasion, and inhibit apoptosis in TGF-β2-induced ARPE-19 cells.

## Discussion

PVR has been confirmed to be a common complication of severe ocular trauma or inflammatory retinopathy [26,27]. At present, PVR is considered to be an excessive injury repair reaction occurring in the eye, but the exact mechanism of its formation is not fully understood. In the past studies, many miRNAs have been confirmed to be related to the development of PVR. For example, Cui *et al*. suggested that miR-194 could inhibit the EMT process in TGF-β1-induced ARPE-19 cells, showing that miR-194 might be a therapeutic target for PVR [23]. Qiang *et al*. reported that miR-34a could suppress ARPE-19 cells proliferation and migration, confirming that miR-34a could serve as a potential target for the prevention of PVR [28]. In addition, miR-29b had been shown to alleviate PVR progression, which could restrain EMT in TGF-β1-stimuluted ARPE-19 cells [29].

In the previously research, miR-4488 was found to exist in extracellular vesicles secreted by breast cancer cells, which could inhibit the angiogenesis of vascular endothelial cells [30]. A recent study indicated that miR-4488 was significantly down-regulated in AAV-infected ARPE-19 cells, which might affect gene targets of multiple biological processes [31]. Here, we investigate the potential role of exosomal miR-4488 in PVR progression. Consistent with the previous study [17,30], our research confirmed that exosomes mediated the intercellular transmission of miR-4488. To confirm the role of exosomal miR-4488 in the EMT of TGF-β2-stimuluted ARPE-19 cells, exosomal miR-4488 was co-cultured with TGF-β2-stimuluted ARPE-19 cells. Function experiments revealed that exosomal miR-4488 suppressed the EMT, migration and invasion, while enhanced the apoptosis of TGF-β2-induced ARPE-19 cells. These data demonstrated that exosomal miR-4488 might be the key miRNAs in mitigating the progression of PVR.

MiR-1273 g has been shown to regulate the progression of many cancer cells, such as colorectal cancer [32] and ovarian cancer [33]. Acute glucose fluctuation could induce endothelial dysfunction by enhancing miR-1273 g expression [34]. Ye *et al*. suggested that miR-1273 g could regulate the autophagy-lysosome pathway to participate in diabetic retinopathy progression [35]. Our data confirmed that miR-1273 g-5p was existed in the exosomes from TGF-β2-induced ARPE-19 cells, which was agreed with the previous research [17]. Overexpressed exosomal miR-1273 g-5p inhibited EMT, migration and invasion in ARPE-19 cells induced by TGF-β2, which confirmed that exosomal miR-1273 g-5p might be a target for PVR treatment.

MiRNAs can bind to the 3ʹUTR of the targets and then instruct the silencing complex to degrade or inhibit the translation of the target mRNA [36,37]. In this, our data revealed that ABCA4 could be targeted by miR-4488 and miR-1273 g-5p. Studies have shown that ABCA4 mutations can cause retinal degenerative diseases, such as Stargardt’s diseases and retinitis pigmentosa [38,39]. The study of Wang *et al*. pointed out the important promoting role of ABCA4 in the development of PVR [21]. Here, we confirmed that ABCA4 reversed the inhibition effect of exosomal miR-4488 and miR-1273 g-5p on EMT, migration and invasion in ARPE-19 cells treated by TGF-β2. These results verified that miR-4488 and miR-1273 g-5p indeed regulated the biological functions of RPE cells by targeting ABCA4. Also, our research further showed that ABCA4 had a promotion effect on the EMT, migration and invasion in TGF-β2-induced ARPE-19 cells, which confirmed the positive role of ABCA4 in PVR progression.

Of course, our current research still has some limitations. Due to experimental conditions and technical limitations, we are temporarily unable to carry out animal experiments to further confirm our conclusions *in vivo*. In future research, we will try to conduct animal experiments to enrich our results.

## Conclusion

In summary, our results indicated that exosomel miR-4488 and miR-1273 g-5p inhibited TGF-β2-induced EMT in ARPE19 cells by targeting ABCA4. These findings revealed that exosomel miR-4488 and miR-1273 g-5p might be the potentially effective targets for the treatment of PVR.

## Highlights

- Exosomal miR-4488 inhibits the EMT of TGF-β2-treated RPE cells.
- Exosomal miR-1273g-5p suppresses the EMT of TGF-β2-treated RPE cells.
- ABCA4 is a target for miR-4488 and miR-1273g-5p.

## References

[1] Pastor JC, Rojas Jimena, Pastor-Idoate S, et al. Proliferative vitreoretinopathy: a new concept of disease pathogenesis and practical consequences. Prog Retin Eye Res. 2016;51:125–155. 26209346 10.1016/j.preteyeres.2015.07.005

[2] Claes C, Lafeta AP. Proliferative vitreoretinopathy. Dev Ophthalmol. 2014;54:188–195. 25196769 10.1159/000360466

[3] Umazume K, Tsukahara R, Liu L, et al. Role of retinal pigment epithelial cell beta-catenin signaling in experimental proliferative vitreoretinopathy. Am J Pathol. 2014;184(5):1419–1428. 24656918 10.1016/j.ajpath.2014.01.022

[4] Chiba C. The retinal pigment epithelium: an important player of retinal disorders and regeneration. Exp Eye Res. 2014;123:107–114. 23880527 10.1016/j.exer.2013.07.009

[5] Zhou M, Geathers JS, Grillo SL, et al. Role of epithelial-mesenchymal transition in retinal pigment epithelium dysfunction. Front Cell Dev Biol. 2020;8:501. 32671066 10.3389/fcell.2020.00501 PMC7329994

[6] Zou H, Shan C, Ma L, et al. Polarity and epithelial-mesenchymal transition of retinal pigment epithelial cells in proliferative vitreoretinopathy. PeerJ. 2020;8:e10136. 33150072 10.7717/peerj.10136 PMC7583629

[7] Mandava N, lackburn P, Paul DB, et al. Ribozyme to proliferating cell nuclear antigen to treat proliferative vitreoretinopathy. Invest Ophthalmol Vis Sci. 2002;43(10):3338–3348. 12356843

[8] Chen N, Hu Z, Yang Y, et al. Inactive Cas9 blocks vitreous-induced expression of Mdm2 and proliferation and survival of retinal pigment epithelial cells. Exp Eye Res. 2019;186:107716. 31278903 10.1016/j.exer.2019.107716

[9] Shukal D, Bhadresha K, Shastri B, et al. Dichloroacetate prevents TGFbeta-induced epithelial-mesenchymal transition of retinal pigment epithelial cells. Exp Eye Res. 2020;197:108072. 32473169 10.1016/j.exer.2020.108072

[10] Ishikawa K, He S, Terasaki H, et al. Resveratrol inhibits epithelial-mesenchymal transition of retinal pigment epithelium and development of proliferative vitreoretinopathy. Sci Rep. 2015;5(1):16386. 26552368 10.1038/srep16386 PMC4639835

[11] Yu X, Odenthal M, Fries JW. Exosomes as miRNA carriers: formation-function-future. Int J Mol Sci. 2016;17:12. 10.3390/ijms17122028 PMC5187828 27918449

[12] Zhang J, Li S, Li L, et al. Exosome and exosomal microRNA: trafficking, sorting, and function. Genomics Proteomics Bioinformatics. 2015;13(1):17–24. 25724326 10.1016/j.gpb.2015.02.001 PMC4411500

[13] Zhu J, Liu B, Wang Z, et al. Exosomes from nicotine-stimulated macrophages accelerate atherosclerosis through miR-21-3p/PTEN-mediated VSMC migration and proliferation. Theranostics. 2019;9(23):6901–6919. 31660076 10.7150/thno.37357 PMC6815950

[14] Sun Z, Shi K, Yang S, et al. Effect of exosomal miRNA on cancer biology and clinical applications. Mol Cancer. 2018;17(1):147. 30309355 10.1186/s12943-018-0897-7 PMC6182840

[15] Qiu L, Wang J, Chen M, et al. Exosomal microRNA146a derived from mesenchymal stem cells increases the sensitivity of ovarian cancer cells to docetaxel and taxane via a LAMC2mediated PI3K/Akt axis. Int J Mol Med. 2020;46(2):609–620. 32626953 10.3892/ijmm.2020.4634 PMC7307828

[16] Zhang L, Li HH, Yuan M, et al. Exosomal miR-22-3p derived from peritoneal macrophages enhances proliferation, migration, and invasion of ectopic endometrial stromal cells through regulation of the SIRT1/NF-kappaB signaling pathway. Eur Rev Med Pharmacol Sci. 2020;24(2):571–580. 32016958 10.26355/eurrev_202001_20033

[17] Zhang Y, Wang K, Pan J, et al. Exosomes mediate an epithelial-mesenchymal transition cascade in retinal pigment epithelial cells: implications for proliferative vitreoretinopathy. J Cell Mol Med. 2020;24(22):13324–13335. 33047885 10.1111/jcmm.15951 PMC7701536

[18] Imani MM, Sadeghi M, Tadakamadla SK, et al. Polymorphisms of ATP-binding cassette, sub-family A, member 4 (rs560426 and rs481931) and non-syndromic cleft Lip/palate: a meta-analysis. Life (Basel). 2021;11:1. 10.3390/life11010058 PMC7830788 33467554

[19] Sung YC, Yang C-H, Yang C-M, et al. Genotypes Predispose Phenotypes-Clinical Features and Genetic Spectrum of ABCA4-Associated Retinal Dystrophies. Genes (Basel). 2020;11:12. 10.3390/genes11121421 PMC7759801 33261146

[20] Holtan JP, Aukrust I, Jansson RW, et al. Clinical features and molecular genetics of patients with ABCA4-retinal dystrophies. Acta Ophthalmol. 2020;99(5):e733-e746. 10.1111/aos.14679 33258285

[21] Wang M, Li Q, Dong H. Proteomic evidence that ABCA4 is vital for traumatic proliferative vitreoretinopathy formation and development. Exp Eye Res. 2019;181:232–239. 30738069 10.1016/j.exer.2019.02.006

[22] Usui-Ouchi A, Ouchi Y, Kiyokawa M, et al. Upregulation of mir-21 levels in the vitreous humor is associated with development of proliferative vitreoretinal disease. PLoS One. 2016;11(6):e0158043. 27351379 10.1371/journal.pone.0158043 PMC4924816

[23] Cui L, Lyu Y, Jin X, et al. miR-194 suppresses epithelial-mesenchymal transition of retinal pigment epithelial cells by directly targeting ZEB1. Ann Transl Med. 2019;7(23):751. 32042767 10.21037/atm.2019.11.90 PMC6990010

[24] Xu J, Liu X, Liu X, et al. Long noncoding RNA KCNMB2-AS1 promotes the development of esophageal cancer by modulating the miR-3194-3p/PYGL axis. Bioengineered. 2021;12(1):6687–6702. 34516362 10.1080/21655979.2021.1973775 PMC8806829

[25] Li X, Li R, Gong Q, et al. Circular RNA circVMA21 ameliorates lipopolysaccharide (LPS)-induced acute kidney injury by targeting the miR-199a-5p/NRP1 axis in sepsis. Biochem Biophys Res Commun. 2021;548:174–181. 33647793 10.1016/j.bbrc.2021.02.028

[26] Dai Y, Dai C, Sun T. Inflammatory mediators of proliferative vitreoretinopathy: hypothesis and review. Int Ophthalmol. 2020;40(6):1587–1601. 32103371 10.1007/s10792-020-01325-4 PMC7242233

[27] Waters T, Vollmer L, Sowka J. Proliferative vitreoretinopathy as a late complication of blunt ocular trauma. Optometry. 2008;79(4):197–202. 18358999 10.1016/j.optm.2007.09.016

[28] Hou Q, Zhou L, Tang J, et al. LGR4 is a direct target of microRNA-34a and modulates the proliferation and migration of retinal pigment epithelial ARPE-19 cells. PLoS One. 2016;11(12):e0168320. 27977785 10.1371/journal.pone.0168320 PMC5158047

[29] Li M, Li H, Liu X, et al. MicroRNA-29b regulates TGF-beta1-mediated epithelial-mesenchymal transition of retinal pigment epithelial cells by targeting AKT2. Exp Cell Res. 2016;345(2):115–124. 25263462 10.1016/j.yexcr.2014.09.026

[30] Zheng X, Lu S, He Z, et al. MCU-dependent negative sorting of miR-4488 to extracellular vesicles enhances angiogenesis and promotes breast cancer metastatic colonization. Oncogene. 2020;39(46):6975–6989. 33067576 10.1038/s41388-020-01514-6

[31] Arumugam S, Mary B, Kumar M, et al. Analysis of hepatic and retinal cell microRNAome during AAV infection reveals their diverse impact on viral transduction and cellular physiology. Gene. 2020;724:144157. 31629820 10.1016/j.gene.2019.144157

[32] Wu F, Liu F, Dong L, et al. miR-1273g silences MAGEA3/6 to inhibit human colorectal cancer cell growth via activation of AMPK signaling. Cancer Lett. 2018;435:1–9. 30056111 10.1016/j.canlet.2018.07.031

[33] Gunel T, Gumusoglu E, Dogan B, et al. Potential biomarker of circulating hsa-miR-1273g-3p level for detection of recurrent epithelial ovarian cancer. Arch Gynecol Obstet. 2018;298(6):1173–1180. 30264202 10.1007/s00404-018-4913-3

[34] Guo J, Sang Y, Yin T, et al. miR-1273g-3p participates in acute glucose fluctuation-induced autophagy, dysfunction, and proliferation attenuation in human umbilical vein endothelial cells. Am J Physiol Endocrinol Metab. 2016;310(9):E734–43. 26908504 10.1152/ajpendo.00444.2015

[35] Ye Z, Li ZH, He SZ. miRNA-1273g-3p involvement in development of diabetic retinopathy by modulating the autophagy-lysosome pathway. Med Sci Monit. 2017;23:5744–5751. 29197896 10.12659/MSM.905336 PMC5724349

[36] Guo H, Ingolia NT, Weissman JS, et al. Mammalian microRNAs predominantly act to decrease target mRNA levels. Nature. 2010;466(7308):835–840. 20703300 10.1038/nature09267 PMC2990499

[37] Agarwal V, Bell GW, Nam Jin-Wu, et al. Predicting effective microRNA target sites in mammalian mRNAs. Elife. 2015;4:e05005 10.7554/eLife.05005 PMC4532895 26267216

[38] Piccardi M, Fadda A, Martelli F, et al. Antioxidant saffron and central retinal function in ABCA4-related stargardt macular dystrophy. Nutrients. 2019;11:10. 10.3390/nu11102461 PMC6835540 31618812

[39] Zhong M, Molday LL, Molday RS. Role of the C terminus of the photoreceptor ABCA4 transporter in protein folding, function, and retinal degenerative diseases. J Biol Chem. 2009;284(6):3640–3649. 19056738 10.1074/jbc.M806580200 PMC4090197

